# Evaluation of the efficacy, safety, and economy of nemonoxacin injection versus moxifloxacin injection in the treatment of community-acquired pneumonia

**DOI:** 10.3389/fphar.2026.1777708

**Published:** 2026-04-29

**Authors:** Yongli Gu, Cong Cheng, Liqun Jiang, Daoli Jiang

**Affiliations:** 1 Department of Pharmacy, The First People’s Hospital of Lianyungang, Lianyungang, Jiangsu, China; 2 School of Pharmacy, Xuzhou Medical University, Xuzhou, Jiangsu, China; 3 Department of Pharmacy, The Affiliated Hospital of Xuzhou Medical University, Xuzhou, China; 4 Jiangsu Key Laboratory of New Drug Research and Clinical Pharmacy, Xuzhou Medical University, Xuzhou, China

**Keywords:** community-acquired pneumonia, cost-minimization analysis, economic evaluation, moxifloxacin, nemonoxacin

## Abstract

**Objective:**

To evaluate the clinical efficacy, safety, and cost-effectiveness of nemonoxacin injection versus moxifloxacin injection in patients with community-acquired pneumonia (CAP), providing an evidence-based reference for clinical decision-making.

**Methods:**

A retrospective cohort study was conducted, enrolling 196 patients hospitalized with CAP between January 2024 and March 2025. Based on the treatment received, patients were allocated to a nemonoxacin group (n = 103) or a moxifloxacin group (n = 93). Clinical efficacy, inflammatory markers before and after treatment, and safety outcomes were compared between the groups. A cost-minimization analysis was used for the economic evaluation.

**Results:**

Baseline characteristics did not differ significantly between groups (*P* > 0.05). Clinical efficacy was 100.0% in the nemonoxacin group versus 97.8% in the moxifloxacin group (*P* = 0.135). Adverse reaction rates were 1.9% and 1.1% for nemonoxacin and moxifloxacin, respectively (*P* = 0.622). Inflammatory markers decreased significantly after treatment in both groups, but the intergroup difference post-treatment was not statistically significant (*P* > 0.05). The total hospitalization cost was significantly lower in the nemonoxacin group (¥6,726.31 ± 1,849.50) than in the moxifloxacin group (¥7,354.12 ± 2,477.16) (*P* = 0.044), indicating superior cost-effectiveness for nemonoxacin. Sensitivity analysis supported the robustness of the economic finding.

**Conclusion:**

Nemonoxacin malate injection demonstrates clinical efficacy and safety comparable to moxifloxacin hydrochloride injection for CAP, while incurring significantly lower treatment costs, resulting in a more favorable economic profile.

## 1 Introduction

Community-acquired pneumonia (CAP) is a prevalent infectious disease, defined as an acute infection of the pulmonary parenchyma acquired outside of hospitals or other healthcare facilities (Gebre et al., 2021). The incidence and mortality of CAP have been rising, with an overall annual incidence estimated at 16–23 cases per 1,000 individuals (Hegde et al., 2022). In the United States alone, the annual healthcare costs associated with CAP can reach $8.4–$12 billion (Amalakuhan et al., 2017; Azeredo da Silveira and Shorr, 2020). CAP is a lower respiratory tract infection caused by various pathogens, including bacteria (e.g., *Streptococcus pneumoniae*, *Haemophilus influenzae*), viruses (e.g., influenza virus, *rhinovirus*), and atypical organisms (e.g., *Legionella*, *Mycoplasma*) (Forastiere, 2024; Khan et al., 2025). Typical clinical manifestations include fever, cough (which may be productive), dyspnea, chest pain, and may be accompanied by systemic symptoms such as fatigue, headache, and myalgia (Ibrahim et al., 2021; Wu et al., 2022). Diagnosis relies on clinical symptoms, laboratory findings (e.g., abnormal white blood cell count and neutrophil percentage, elevated C-reactive protein or procalcitonin suggesting bacterial infection), and chest imaging (e.g., consolidation or multi-lobar infiltrates on X-ray or CT) (Hansen et al., 2023).

Compared to other antimicrobial classes (e.g., penicillins, cephalosporins, macrolides, β-lactams), the newer quinolones are currently the only ideal agents that can be used as monotherapy to effectively cover both extracellular typical bacteria and intracellular atypical pathogens. Their monotherapy efficacy is comparable to β-lactam/macrolide combinations and has been associated with significantly reduced mortality in severe CAP patients (M’Ikanatha et al., 2021; Wu et al., 2021). Nemonoxacin and moxifloxacin are commonly used quinolone antibiotics for CAP. They share a similar bactericidal mechanism, targeting bacterial DNA gyrase and topoisomerase IV, leading to irreversible DNA damage and inhibition of bacterial growth (Huang et al., 2015; Li et al., 2010). However, they differ in structure, antibacterial spectrum, pharmacokinetics, and clinical application profiles. Nemonoxacin is a non-fluorinated quinolone with a long half-life and concentration-dependent killing. It exhibits potent activity against methicillin-resistant *Staphylococcus aureus* (MRSA) and a potentially lower risk of toxicity, suggesting advantages in safety (Cao et al., 2014; Khan et al., 2025; Yuan et al., 2022). Moxifloxacin, a representative fourth-generation fluoroquinolone, possesses a broad spectrum and potent activity against common pathogens like *S. pneumoniae*, *Mycoplasma*, and *Chlamydia*, along with higher anti-anaerobic activity and excellent lung tissue penetration, making it particularly suitable for complicated CAP (Dandagi et al., 2013).

While both nemonoxacin and moxifloxacin are widely used for CAP treatment, systematic evaluations directly comparing their efficacy, safety, and economic impact remain limited. We hypothesized that nemonoxacin would demonstrate comparable efficacy and safety to moxifloxacin, with a potentially more favorable economic profile, positioning this study as an exploratory comparison rather than a formal superiority or non-inferiority assessment. Therefore, this study aims to perform a comparative assessment of nemonoxacin injection and moxifloxacin injection in CAP treatment regarding clinical efficacy, safety, and economy, thereby providing more robust evidence to guide clinical practice.

## 2 Materials and methods

### 2.1 Data source and study design

This retrospective cohort study screened and enrolled 196 patients hospitalized with CAP at Lianyungang First People’s Hospital, Jiangsu Province, China, between January 2024 and March 2025. Patients received intravenous infusions of either nemonoxacin malate injection (0.5 g once daily; Zhejiang Medicine Co., Ltd., Xinchang Pharmaceutical Factory) or moxifloxacin hydrochloride injection (0.4 g once daily; Yangtze River Pharmaceutical Group Co., Ltd.). Based on the administered medication, patients were categorized into the Nemonoxacin group (n = 103) and the Moxifloxacin group (n = 93). Patient data were collected from the hospital information management system.

### 2.2 Inclusion and exclusion criteria

Inclusion criteria: (1) Diagnosis of CAP meeting the criteria outlined in the “Diagnosis and Treatment of Adults with Community-acquired Pneumonia” (Metlay et al., 2019), confirmed by chest X-ray and laboratory tests; (2) Age between 18 and 80 years, either gender; (3) No prior use of antibiotics before hospitalization. Exclusion criteria: (1) Treatment duration less than 5 days or greater than 14 days; (2) Patients with concomitant malignancies, respiratory failure, or other severe conditions; (3) History of allergy to the study medications.

### 2.3 Observation indicators

Efficacy assessment timepoint and criteria: Efficacy was assessed at the end of treatment (EOT), defined as the day of discharge or the day of intravenous antibiotic discontinuation. For patients with treatment duration exceeding 7 days, an additional assessment was performed at Day 7 to ensure consistency. The following definitions, adapted from the ATS/IDSA CAP guidelines (Metlay et al., 2019), were used: (1) Clinical cure: Complete resolution or return to baseline of all signs and symptoms of pneumonia, with no need for additional antibiotic therapy; (2) Clinical improvement: Partial resolution of signs and symptoms, not requiring a change in antibiotic class; (3) Treatment failure: Persistence or worsening of signs and symptoms requiring a change in antibiotic therapy. For the purpose of this study, “Effective” was defined as clinical cure or clinical improvement. Efficacy assessment was performed by the treating physicians as part of routine clinical care, not by independent blinded assessors, which is an inherent limitation of retrospective studies.

Disease severity assessment: Disease severity at admission was assessed using the CURB-65 score (Metlay et al., 2019). This score comprises five components, each assigned one point: (1) Confusion (new-onset disorientation to person, place, or time); (2) Blood urea nitrogen >7 mmol/L; (3) Respiratory rate ≥30 breaths/min; (4) Hypotension (systolic blood pressure <90 mmHg or diastolic blood pressure ≤60 mmHg); (5) Age ≥65 years. The total score ranges from 0 to 5, with higher scores indicating greater disease severity. All components were extracted from electronic medical records within 24 h of admission.

(1) Adverse Reactions (ADRs): Documented ADRs included common ones (e.g., rash, headache, dizziness, insomnia) and serious reactions (e.g., QTc prolongation, tendon injury, blood glucose abnormalities, peripheral neuropathy). (2) Temperature and Inflammatory Markers: Body temperature was recorded, and venous blood was drawn after fasting before and after treatment to measure White Blood Cell count (WBC), C-Reactive Protein (CRP) level, and Neutrophil Percentage (N%). (3) Costs: Data obtained from the hospital records included total hospitalization cost, total drug cost, antimicrobial drug cost, diagnostic fees, nursing fees, treatment fees, material costs, and other expenses. Indirect and intangible costs were not considered due to the retrospective design.

### 2.4 Evaluation methods

As a retrospective cohort study without randomization, baseline characteristics were first compared. If no significant differences existed, outcomes were compared directly. If significant differences were found, propensity score matching would be employed to control for confounding variables. Given comparable clinical efficacy and safety outcomes between groups, a cost-minimization analysis (CMA) was used for the economic evaluation. Total hospitalization cost was pre-specified as the primary economic endpoint.

### 2.5 Sensitivity analysis

Since cost data were not normally distributed, a Generalized Linear Model (GLM) with a Gamma distribution and Identity link function was used, with total hospitalization cost as the dependent variable. The model included only baseline clinical and demographic covariates (age, gender, comorbidities, CURB-65 score, glucocorticoid use, and additional antibiotic use). Furthermore, bootstrap resampling (5,000 repetitions) was performed to construct a cost-effectiveness acceptability curve (CEAC), accounting for uncertainty in sampling and the distribution of cost data.

### 2.6 Statistical analysis

A *post hoc* power calculation was performed for the primary endpoint of total hospitalization cost using G*Power 3.1 software (Heinrich-Heine-Universität Düsseldorf, Düsseldorf, Germany), with parameters including the observed mean difference, pooled standard deviation, and sample sizes of the two groups. All *P*-values are reported as exact values, with the notation *P* < 0.001 accepted where applicable.

Statistical analyses were performed using SPSS Statistics 30. Normally distributed continuous data are presented as mean ± standard deviation (SD) and compared using the t-test. Non-normally distributed data are presented as median (interquartile range) and compared using the Mann-Whitney U test. For cost comparisons, independent-samples t-tests were used, justified by the Central Limit Theorem given the sample sizes (n > 90 in both groups). Categorical data are expressed as counts (percentages) and compared using the Pearson chi-square test. A *P*-value < 0.05 was considered statistically significant.

## 3 Results

### 3.1 Baseline characteristics of patients

A total of 196 eligible CAP patients were included and divided into the Nemonoxacin group (n = 103) and the Moxifloxacin group (n = 93). As shown in Table 1, no statistically significant differences were observed between the two groups regarding age, gender, treatment duration, comorbidities (hypertension, diabetes, cerebral infarction), CURB-65 score, use of glucocorticoids, or additional use of one or two other antimicrobial agents (all *P* > 0.05). This indicates that baseline characteristics were balanced and unlikely to confound the comparisons of efficacy, safety, and economy.

**TABLE 1:** Comparison of baseline characteristics between the two groups.

Characteristic	Nemonoxacin group (n = 103)	Moxifloxacin group (n = 93)	Statistical value (t/χ^2^/Z)	*P*-value
Age (years), mean ± SD	53.69 ± 17.90	58.48 ± 18.05	t = −1.865	0.064
Male, n (%)	53 (51.5%)	54 (58.1%)	χ^2^ = 0.861	0.353
Treatment duration (days), mean ± SD	7.35 ± 2.14	7.87 ± 2.86	t = −1.451	0.148
Comorbidities, n (%)				
Hypertension	17 (16.5%)	22 (23.7%)	χ^2^ = 1.568	0.210
Diabetes	6 (5.8%)	10 (10.8%)	χ^2^ = 1.583	0.208
Cerebral infarction	0 (0.0%)	2 (2.2%)	χ^2^ = 2.238	0.135
CURB-65 score, median (IQR)	1.00 (1.00,2.00)	1.00 (1.00,2.00)	Z = −0.080	0.936
CURB-65 components, n (%)				
Confusion, n (%)	0 (0.0%)	0 (0.0%)	—	—
BUN > 7 mmol/L, n (%)	8 (7.8%)	9 (9.7%)	χ^2^ = 0.226	0.634
Respiratory rate ≥30/min, n (%)	3 (2.9%)	4 (4.3%)	χ^2^ = 0.288	0.591
Hypotension, n (%)	2 (1.9%)	1 (1.1%)	χ^2^ = 0.243	0.622
Age ≥65 years, n (%)	28 (27.2%)	32 (34.4%)	χ^2^ = 1.220	0.269
Additional antimicrobials, n (%)				
1 additional agent	51 (49.5%)	45 (48.4%)	χ^2^ = 0.025	0.875
2 additional agents	25 (24.3%)	23 (24.7%)	χ^2^ = 0.006	0.940
Glucocorticoid use, n (%)	62 (60.2%)	47 (50.5%)	χ^2^ = 1.846	0.174

SD, standard deviation; IQR, interquartile range; CURB-65, Confusion, Urea, Respiratory rate, Blood pressure, age 65; BUN, blood urea nitrogen.

### 3.2 Comparison of clinical efficacy

The total effectiveness rate was 100.0% in the Nemonoxacin group and 97.8% in the Moxifloxacin group. This difference was not statistically significant (*P* > 0.05), as shown in Table 2. Both drugs demonstrated high efficacy in treating CAP.

**TABLE 2:** Comparison of clinical efficacy between the two groups [n (%)].

Group	Effective	Ineffective	χ^2^	*P*-value
Nemonoxacin (n = 103)	103 (100.0%)	0 (0.0%)	2.238	0.135
Moxifloxacin (n = 93)	91 (97.8%)	2 (2.2%)	​	​

### 3.3 Comparison of Adverse Reactions

The incidence of ADRs was 1.9% (2 cases: cough, rash) in the Nemonoxacin group and 1.1% (1 case: arthralgia in limbs) in the Moxifloxacin group. The difference was not statistically significant (*P* > 0.05, Table 3). Both drugs exhibited similarly low ADR rates. All reported ADRs were mild and resolved spontaneously without incurring additional costs.

**TABLE 3:** Comparison of adverse drug reaction incidence between the two groups [n (%)].

Group	With ADRs	Without ADRs	χ^2^	*P*-value
Nemonoxacin (n = 103)	2 (1.9%)	101 (98.1%)	0.243	0.622
Moxifloxacin (n = 93)	1 (1.1%)	92 (98.9%)	​	​

### 3.4 Comparison of temperature and inflammatory markers before and after treatment

Body temperature decreased significantly after treatment in both groups (*P* < 0.05). Neutrophil percentage (N%) and CRP levels also showed significant reductions from pre-to post-treatment in both groups (*P* < 0.05), as detailed in Tables 4, 5. However, when comparing the two groups after treatment, no statistically significant differences were found in body temperature, WBC, N%, or CRP levels (all *P* > 0.05, Table 6).

**TABLE 4:** Comparison of temperature and inflammatory markers before and after treatment in the Nemonoxacin group (mean ± SD).

Timepoint	Temperature (°C)	WBC (×10^9^/L)	N (%)	CRP (mg/L)
Pre-treatment	37.94 ± 1.18	8.00 ± 3.88	69.71 ± 16.46	53.84 ± 45.97
Post-treatment	36.45 ± 0.19	7.37 ± 3.35	64.10 ± 13.99	13.24 ± 24.88
*P*-value	<0.001	0.119	<0.001	<0.001

The post-treatment values presented in this table represent paired data from patients with complete pre- and post-treatment records. Therefore, the sample size for post-treatment analysis in Table 4 may differ from that in Table 6, which includes all available post-treatment measurements regardless of paired data availability. WBC, white blood cell; N, neutrophil; CRP, C-reactive protein; SD, standard deviation.

**TABLE 5:** Comparison of temperature and inflammatory markers before and after treatment in the Moxifloxacin group (mean ± SD).

Timepoint	Temperature (°C)	WBC (×10^9^/L)	N (%)	CRP (mg/L)
Pre-treatment	37.87 ± 1.19	8.12 ± 3.64	71.93 ± 13.83	60.89 ± 51.01
Post-treatment	36.48 ± 0.18	7.46 ± 3.59	65.78 ± 12.42	18.99 ± 31.03
*P*-value	<0.001	0.252	0.017	<0.001

The post-treatment values presented in this table represent paired data from patients with complete pre- and post-treatment records. Therefore, the sample size for post-treatment analysis in Table 5 may differ from that in Table 6, which includes all available post-treatment measurements regardless of paired data availability. WBC, white blood cell; N, neutrophil; CRP, C-reactive protein; SD, standard deviation.

**TABLE 6:** Comparison of temperature and inflammatory markers between the two groups after treatment (mean ± SD).

Group	Temperature (°C)	WBC (×10^9^/L)	N (%)	CRP (mg/L)
Nemonoxacin (n = 103)	36.44 ± 0.19	7.15 ± 3.02	62.77 ± 15.29	7.71 ± 13.85
Moxifloxacin (n = 93)	36.48 ± 0.18	7.52 ± 3.52	66.51 ± 12.50	18.80 ± 30.13
*P*-value	0.168	0.581	0.200	0.052

The post-treatment values presented in this table include all available measurements from patients who had post-treatment data, regardless of whether paired pre-treatment data were available. This explains the slight discrepancies between the post-treatment values reported in Tables 4–6. Sample sizes for specific parameters may vary due to missing data. WBC, white blood cell; N, neutrophil; CRP, C-reactive protein; SD, standard deviation.

### 3.5 Cost-minimization analysis

Given the comparable clinical efficacy and safety profiles, a cost-minimization analysis was conducted. As presented in Table 7, the total hospitalization cost was significantly lower in the Nemonoxacin group (¥6,726.31 ± 1,849.50) than in the Moxifloxacin group (¥7,354.12 ± 2,477.16) (*P* < 0.05). Diagnostic fees and material costs were also significantly lower in the Nemonoxacin group. However, the antimicrobial drug cost was significantly higher in the Nemonoxacin group (¥945.35 ± 794.06 vs. ¥626.04 ± 800.37, *P* < 0.05), attributable to the higher unit price of Nemonoxacin. No significant differences were found in other cost categories. Therefore, with equivalent effectiveness and safety, nemonoxacin injection demonstrated superior cost-effectiveness.

**TABLE 7:** Comparison of direct medical costs between the two groups (unit: Chinese Yuan, mean ± SD).

Cost category	Nemonoxacin group (n = 103)	Moxifloxacin group (n = 93)	*P*-value
Total hospitalization cost	6726.31 ± 1849.50	7354.12 ± 2477.16	0.044
Total drug cost	1875.27 ± 1328.33	1857.03 ± 1688.18	0.933
Antimicrobial drug cost	945.35 ± 794.06	626.04 ± 800.37	0.006
Diagnostic fees	3354.40 ± 1119.46	3750.81 ± 1275.17	0.022
Nursing fees	341.46 ± 148.22	384.32 ± 163.20	0.055
Treatment fees	982.35 ± 989.80	1204.14 ± 868.31	0.099
Material costs	117.68 ± 101.01	199.70 ± 295.77	0.004
Other costs	390.37 ± 234.02	408.15 ± 255.74	0.612

SD, standard deviation.

Post-hoc power analysis for the primary cost endpoint: Based on the observed mean difference in total hospitalization cost (¥627.81), pooled standard deviation (¥2,186.0), and sample sizes (n = 103 and n = 93), a *post hoc* power calculation using G*Power 3.1 software revealed that the study had 84% power to detect this difference at a two-sided alpha level of 0.05.

### 3.6 Uncertainty analysis

#### 3.6.1 Generalized linear model (GLM) for costs

A GLM analysis with total hospitalization cost as the dependent variable, adjusting for baseline clinical and demographic covariates, confirmed that the choice of drug (“Medication”) significantly influenced the total cost (*P* < 0.05), with nemonoxacin associated with lower costs, consistent with the primary analysis (Table 8).

**TABLE 8:** Generalized Linear Model regression analysis of total hospitalization costs.

Parameter	B coefficient	SE	95% CI lower	95% CI upper	Wald χ^2^	df	*P*-value
(Intercept)	6128.345	892.456	4379.567	7877.123	47.156	1	<0.001
Medication (nemo vs. moxi)	−270.600	150.514	−550.323	−3.998	7.957	1	0.045
Age	15.234	12.567	−9.398	39.866	1.469	1	0.225
Gender	−89.456	298.789	−675.123	496.211	0.090	1	0.765
Hypertension	−212.567	345.678	−890.123	464.989	0.378	1	0.539
Diabetes	134.789	423.456	−695.123	964.701	0.101	1	0.750
Cerebral infarction	567.234	867.890	−1133.567	2268.035	0.427	1	0.513
Extra antibiotic (1)	223.456	312.567	−389.123	836.035	0.511	1	0.475
Extra antibiotic (2)	445.678	389.123	−317.567	1208.923	1.312	1	0.252
Glucocorticoid use	−6.462	150.900	−780.245	−290.679	20.048	1	<0.001
CURB-65 score	345.678	223.456	−92.345	783.701	2.394	1	0.122

Categorical variables were coded as follows: Medication (0 = Moxifloxacin, 1 = Nemonoxacin); Gender (0 = Female, 1 = Male); Hypertension, Diabetes, Cerebral Infarction, Extra Antibiotic (1 agent), Extra Antibiotic (2 agents), Glucocorticoid Use (0 = No, 1 = Yes). CI, Confidence Interval; df, Degrees of Freedom; CURB-65, Confusion, Urea, Respiratory rate, Blood pressure, age 65; SE, Standard Error.

#### 3.6.2 Bootstrap analysis

**FIGURE 1:**
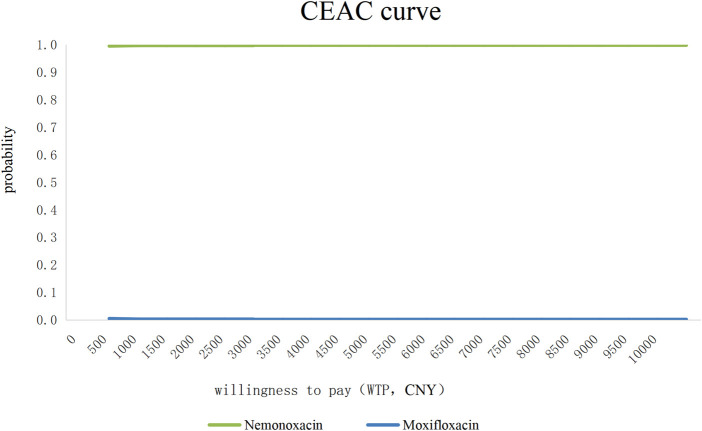
Cost-effectiveness acceptability curve from Bootstrap analysis.

Bootstrap resampling (5,000 repetitions) with treatment effectiveness as the outcome and total cost as the cost indicator was performed. The resulting cost-effectiveness acceptability curve (CEAC, Figure 1) demonstrated that the probability of nemonoxacin being the more cost-effective option exceeded 99% across a wide range of willingness-to-pay thresholds (¥0 to ¥10,000), robustly supporting the primary finding.

## 4 Discussion

Although treatment allocation was not randomized in this retrospective study, baseline characteristics between the two groups were statistically comparable. To further mitigate potential selection bias, key demographic and clinical variables—including age, gender, comorbidities, and concomitant medication use—were incorporated as covariates in a Generalized Linear Model. The results confirmed that the choice of antimicrobial agent remained a significant independent predictor of total hospitalization cost (*P* < 0.05), supporting the robustness of the primary findings. Nevertheless, the possibility of unmeasured confounding cannot be entirely excluded, and future prospective randomized controlled trials are warranted to validate these results.

This retrospective cohort study demonstrates that nemonoxacin injection is comparable to moxifloxacin injection in terms of clinical efficacy and safety for the treatment of CAP but offers a clear economic advantage through lower total hospitalization costs. It should be noted that formal equivalence or non-inferiority testing was not performed in this study; therefore, the cost-minimization analysis relies on the assumption of comparable efficacy and safety rather than formal proof. Future prospective trials with pre-specified non-inferiority margins are warranted to confirm these findings.

Our findings of comparable high clinical efficacy (100% vs. 97.8%, *P* > 0.05) align with the known potent antibacterial activity of both drugs against common CAP pathogens (Kocsis et al., 2016; Zhao et al., 2022). The observed clinical efficacy rates—100% in the nemonoxacin group and 97.8% in the moxifloxacin group—are higher than those typically reported in large-scale clinical trials. This may be attributed to the study’s inclusion of patients with mild-to-moderate CAP, the absence of microbiologically confirmed refractory cases, and the relatively lenient definition of “effective” based on clinical and radiological improvement. These factors should be considered when interpreting the generalizability of the efficacy outcomes, particularly to populations with severe CAP or those caused by drug-resistant pathogens.

It is important to note that microbiological data, including pathogen identification and susceptibility testing, were not available for the majority of patients in this retrospective cohort. Consequently, the correlation between clinical response and microbiological eradication could not be assessed. Although both agents exhibit broad-spectrum activity against typical and atypical CAP pathogens, the absence of microbiological confirmation limits the ability to attribute clinical outcomes directly to antimicrobial efficacy. Future studies should incorporate microbiological endpoints to strengthen causal inferences.

The similar significant reductions in inflammatory markers (WBC, N%, CRP) in both groups further corroborate their comparable clinical effectiveness, as these markers are well-established indicators of systemic inflammation and treatment response in CAP (Yang et al., 2020). It should be noted that the post-treatment values presented in Table 6 differ slightly from those in Tables 4, 5. This is because Tables 4, 5 display paired pre- and post-treatment data only for patients with complete records, whereas Table 6 includes all available post-treatment measurements, regardless of whether paired pre-treatment data were available. This discrepancy is clearly indicated in the table footnotes to ensure transparency in data presentation. The anti-inflammatory properties intrinsic to quinolones, potentially mediated through immunomodulation and effects on cAMP signaling and cytokine production (e.g., IL-6, TNF-α), might contribute to this observed effect (Chifiriuc et al., 2011; Krehmeier et al., 2002; Liu et al., 2018).

The safety profiles of both drugs were favorable and statistically indistinguishable (ADR rate 1.9% vs. 1.1%, *P* > 0.05). The adverse events reported were mild and self-limiting. While fluoroquinolones like moxifloxacin carry known class warnings (e.g., QTc prolongation, tendonitis), these serious events were not observed in our cohort, possibly due to the study’s sample size and patient selection. The non-fluorinated structure of nemonoxacin may theoretically confer a lower risk for certain fluoroquinolone-associated adverse effects (Khan et al., 2023; Li et al., 2024; Qin and Huang, 2014), a potential benefit that requires confirmation in larger-scale studies. The incidence of adverse drug reactions reported in this study (1.9% for nemonoxacin and 1.1% for moxifloxacin) is substantially lower than that documented in prospective clinical trials of quinolones, where rates typically range from 10% to 30%. This discrepancy is likely attributable to the inherent limitations of retrospective data collection, including under documentation in medical records and the absence of active surveillance for adverse events. Therefore, the safety profiles observed here should be interpreted with caution, and prospective pharmacovigilance studies are needed to comprehensively evaluate the safety of nemonoxacin in real-world settings.

The core finding of this study lies in the economic evaluation. Despite a significantly higher drug acquisition cost for nemonoxacin, the total hospitalization cost was substantially lower in the nemonoxacin group. This suggests that the initial higher drug cost was offset by reductions in other cost components, notably diagnostic fees and material costs. The reasons for these lower associated costs are likely multifactorial and may relate to more rapid clinical stabilization, potentially leading to less intensive diagnostic monitoring or utilization of consumables, though our study design cannot definitively establish causality. The lower treatment and material costs observed in the nemonoxacin group, despite comparable treatment duration and hospital stay, may suggest a faster clinical stabilization leading to reduced utilization of diagnostic tests and consumables. However, due to the retrospective design, it was not possible to establish a causal relationship between drug choice and specific cost components. Future prospective studies should collect detailed resource utilization data to elucidate the drivers of cost differences between these two antimicrobial regimens.

Disease severity is a critical determinant of clinical outcomes and healthcare costs in patients with CAP. In this study, we successfully calculated the complete CURB-65 score for all 196 patients based on the five standard components extracted from electronic medical records. The CURB-65 score was 1.00 (IQR: 1.00–2.00) in both groups, with no statistically significant difference (*P* = 0.936). The low CURB-65 scores in both groups (median 1.00) confirm that the study population consisted predominantly of low-risk, mild-to-moderate CAP patients. These findings demonstrate that disease severity was well-balanced between the two treatment groups, effectively addressing this potential confounder.

Although the difference in post-treatment C-reactive protein (CRP) levels between the two groups did not reach statistical significance (*P* = 0.052), the numerical difference (7.71 mg/L in the nemonoxacin group vs. 18.80 mg/L in the moxifloxacin group) is clinically noteworthy. This trend may reflect a more rapid resolution of inflammation or enhanced infection control in patients treated with nemonoxacin. However, given the variability in individual responses and the sample size, this finding should be interpreted as hypothesis-generating and warrants further investigation in larger, prospective cohorts. Regarding the missing data pattern for inflammatory markers, complete paired pre- and post-treatment CRP data were available for 76 of 103 patients (73.8%) in the nemonoxacin group and 71 of 93 patients (76.3%) in the moxifloxacin group. Patients with missing pre-treatment CRP values were predominantly those with mild symptoms who were discharged early (within 24–48 h), before routine follow-up CRP testing was performed. Therefore, the missing pattern was not completely random but was associated with milder disease and shorter hospital stays. Because the proportion of missing data was similar between groups (approximately 25% in both), the between-group comparisons remain valid for the primary cost and efficacy analyses.

This result is consistent with the findings of Zhao et al., who also reported nemonoxacin to be the most cost-effective option among quinolones for CAP (Zhao et al., 2022). This economic advantage was further supported by sensitivity analyses. The robustness of our economic conclusion is strongly supported by both one-way sensitivity analysis using GLM, which identified “Medication” as a significant independent predictor of total cost, and probabilistic sensitivity analysis using bootstrap resampling, which showed a greater than 99% probability of nemonoxacin being cost-effective across a wide range of willingness-to-pay thresholds. The cost-effectiveness acceptability curve derived from bootstrap resampling demonstrated a near-100% probability that nemonoxacin is cost-effective across all willingness-to-pay thresholds. This atypical flat-line pattern reflects the dominance of nemonoxacin over moxifloxacin in the economic evaluation, driven by significantly lower total hospitalization costs despite higher drug acquisition costs. The robustness of this finding was confirmed by repeated bootstrap sampling and net benefit regression analysis, which are appropriate methodologies for assessing uncertainty in cost-effectiveness analyses with dominant strategies.

Several limitations of this study should be acknowledged. First, the retrospective, single-center design inherently limits the generalizability of the findings and may introduce assessment bias, as efficacy assessment was performed by treating physicians rather than independent blinded assessors. Second, while the *post hoc* power calculation confirmed adequate power (84%) to detect the primary cost difference, the study may be underpowered for secondary outcomes such as rare adverse events (expected incidence < 2%); therefore, these findings should be interpreted with caution. Third, the specific reasons for the observed differences in diagnostic and material costs warrant further investigation in prospective studies designed to capture detailed resource utilization data. Fourth, the cost data are derived from a single Chinese hospital; thus, the absolute cost differences may not be directly translatable to other healthcare systems, although the relative economic advantage of nemonoxacin is a significant finding. Finally, the absence of microbiological data and the reliance on the assumption of comparable efficacy and safety (rather than formal non-inferiority testing) for the cost-minimization analysis represent additional limitations that should be considered when interpreting the results. Future prospective, multicenter, randomized controlled trials with pre-specified non-inferiority margins and comprehensive microbiological and resource utilization data are warranted to validate these findings.

## 5 Conclusion

In conclusion, nemonoxacin malate injection demonstrates clinical efficacy and safety equivalent to moxifloxacin hydrochloride injection for the treatment of CAP. Crucially, treatment with nemonoxacin was associated with significantly lower total hospitalization costs, making it a more economically efficient choice. Therefore, nemonoxacin presents itself as a valuable therapeutic option for CAP, worthy of broader clinical adoption based on its favorable economic profile alongside robust efficacy and safety.

## Data Availability

The original contributions presented in the study are included in the article/supplementary material, further inquiries can be directed to the corresponding author.
